# Prospective motor control obeys to idiosyncratic strategies in autism

**DOI:** 10.1038/s41598-018-31479-2

**Published:** 2018-09-12

**Authors:** Andrea Cavallo, Luca Romeo, Caterina Ansuini, Jessica Podda, Francesca Battaglia, Edvige Veneselli, Massimiliano Pontil, Cristina Becchio

**Affiliations:** 1 Department of Psychology, University of Torino, Torino, Italy; 2 Cognition, Motion & Neuroscience Unit, Fondazione Istituto Italiano di Tecnologia, Genova, Italy; 3 Computational Statistics and Machine Learning, Fondazione Istituto Italiano di Tecnologia, Genova, Italy; 4 Dipartimento di Ingegneria dell’Informazione, Università Politecnica delle Marche, Ancona, Italy; 5 Child Neuropsychiatric Unit, G. Gaslini Institute, Genoa, Italy; 6 Department of Computer Science, University College London, London, UK

## Abstract

Disturbance of primary *prospective motor control* has been proposed to contribute to faults in higher mind functions of individuals with autism spectrum disorder, but little research has been conducted to characterize prospective control strategies in autism. In the current study, we applied pattern-classification analyses to kinematic features to verify whether children with autism spectrum disorder (ASD) and typically developing (TD) children altered their initial grasp in anticipation of self- and other-actions. Results indicate that children with autism adjusted their behavior to accommodate onward actions. The way they did so, however, varied idiosyncratically from one individual to another, which suggests that previous characterizations of general lack of prospective control strategies may be overly simplistic. These findings link abnormalities in anticipatory control with increased variability and offer insights into the difficulties that individuals with ASD may experience in social interaction.

## Introduction

The simple act of picking up a water glass is the product of multilayered cognitive plans and sophisticated neural computations^1,2^. At the heart of these computations is *prediction*: motor performance anticipates futures states. This goes beyond anticipating the properties of the object being reached for^3,4^. People alter their manipulative behavior in anticipation of what they plan to do *next* with the object, e.g. if they plan to drink from the glass or pass the glass to another person^5^. Moreover, during joint actions, they may alter their initial grasp to accommodate the onward actions of others. That is, they alter their grasp based on what they expect their partner will do next with the object^6^.

Failure to develop this primary form of *prospective motor control* has been proposed to contribute to faults in higher mind functions of individuals with autism spectrum disorder (ASD)^7,8^. Yet, the very notion of abnormalities in the prospective motor control in autism remains controversial. Empirical support is mixed and interpretations are varied, potentially because no common pattern characterizes ASD individuals homogeneously.

Heterogeneity in individual responses is a consistent finding in autism research^9–12^ and has been proposed to reflect a distinctive characteristic of neural activity in ASD^13^. However, to date, no study has assessed its impact on prospective motor control. In the current study, we introduce a novel approach for parsing this heterogeneity through machine learning modeling of the kinematics of children with ASD and typically developing (TD) children performing a sequential object manipulation task.

Computationally, the task of uncovering *prospective* control strategies governing manipulative behavior can be framed as a pattern-classification problem. Specifically, can the way in which an object is grasped reveal the action to be performed next? Our approach draws on ideas from pattern-classification for analyzing subtle changes in kinematics as spatiotemporal patterns and linking them to the forthcoming action, be it self- or other-performed. By applying machine learning methods to movement features, we first assessed the extent to which children in the ASD group prospectively altered their manipulative behavior in comparison to children in the group. Using *multivariate cross-classification*^14,15^, we next investigated the correspondence between prospective control strategies across ASD and TD groups. Finally, we quantified individual pattern distortions within each group and correlation with ASD symptoms.

This multilevel pattern-classification approach was applied to test prospective control to accommodate both one’s own and another person’s action plans. Observations of grasping suggest that while 3-years-old TD children are already able to plan self-actions in advance, development of prospective of other-actions is protracted during middle childhood, starting to emerge around 7 years of age^16^. Evidence of similar developmental timeline in ASD to date has been sparse and inconsistent^17,18^, which again may be a consequence of variability among individuals with autism. Consistent with this notion, our results reveal a marked heterogeneity within the ASD prospective control strategies, suggesting that ASD children vary idiosyncratically in the ways they alter their grasp to accommodate self- and other-actions.

## Results

We used a sequential object manipulation task to test prospective motor control in children with ASD (*n* = 20) and IQ-matched typically developing (TD) children (*n* = 20). Children were instructed to reach towards and grasp an object (a bottle), to place it into a box (*grasp-to-place*), to pour some water into a glass (*grasp-to-pour*), or to pass the bottle to a co-actor (*grasp-to-pass*), who would then either place the bottle into the box (*pass-to-place*) or pour some water (*pass-to-pour*).

A near-infrared camera motion capture system was used to record movement kinematics. Kinematic parameters of interest (*N* = 15, see ‘Kinematics recording and data processing’ section) were computed throughout the reach-to-grasp phase of the movement (from reach onset to the moment of contact between the fingers and the bottle, i.e., grasp offset. See Supplementary Videos 1–8) at intervals of 1% of the normalized movement time. The 1500 resulting features were used as predictors for all the classification analyses.

### Prospective control of self-actions

**Figure 1:**
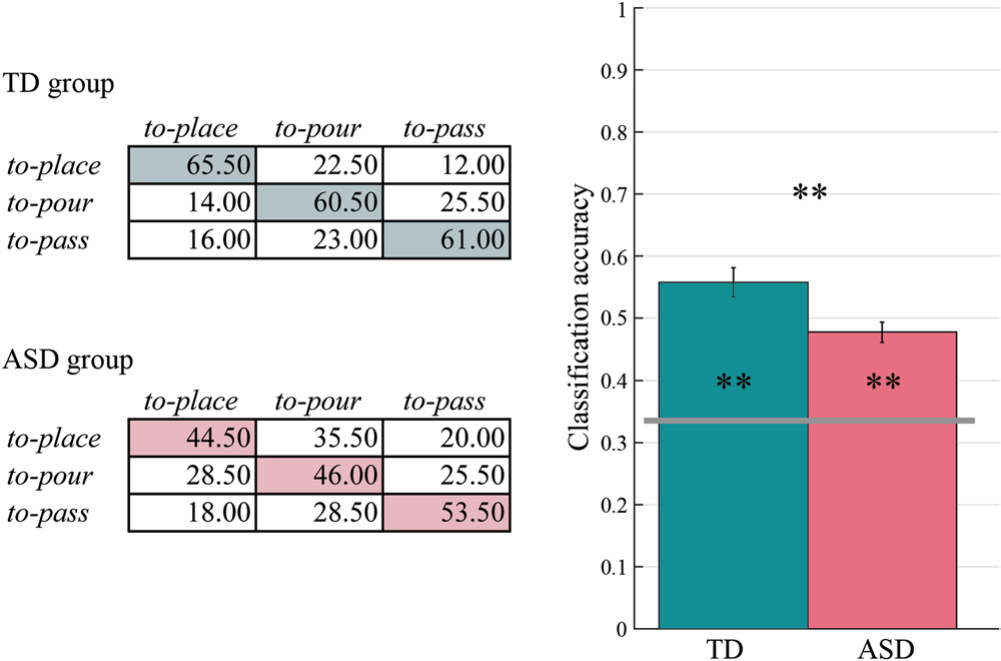
Prospective control of self-actions. Confusion matrices and classification accuracies for TD and ASD group. The classification accuracy exceeded chance level (0.33; grey horizontal line) in both groups (empirical *p*_*s*_ after 500 permutations = 0.002) but was significantly lower in ASD compared to TD group (*p* < 0.01). Asterisks inside bars indicate significant differences from chance level classification. Asterisk outside bars indicates significant differences between groups (**p < 0.01).

To quantify changes in behavior as a function of self-action plans, we first trained a Gaussian Kernel support vector machine (SVM) to distinguish, separately for the ASD and the TD group, grasping movements followed by one of three onward self-actions: *place*, *pour*, and *pass*. Classification accuracy, computed as the resulting average of a leave-one-subject-out cross-validation procedure, was used as a measure of classifier performance. To test whether *classification performance* significantly exceeded chance level, we randomly permuted the class labels ‘*place’*, ‘*pour’*, and ‘*pass’* (500 permutations) and recomputed SVMs *classification accuracy* after each permutation. Classifier performance exceeded chance level (0.33) in both the TD group (mean = 0.623, 95% CI = [0.559, 0.687], empirical *p* after 500 permutations = 0.002) and the ASD group (mean = 0.480, 95% CI = [0.415, 0.545], empirical *p* = 0.002). This demonstrates that, in both groups, forthcoming self-demands resulted in anticipatory modifications of the initial grasping. Tailoring to the onward self-action was less pronounced in the ASD group than in the TD group as indicated by a lower *classification accuracy* (independent samples t-test, t_38_ = −3.285, *p* < 0.01). Figure 1 shows confusion matrices and *classification accuracies* for each of the two groups.

**Figure 2:**
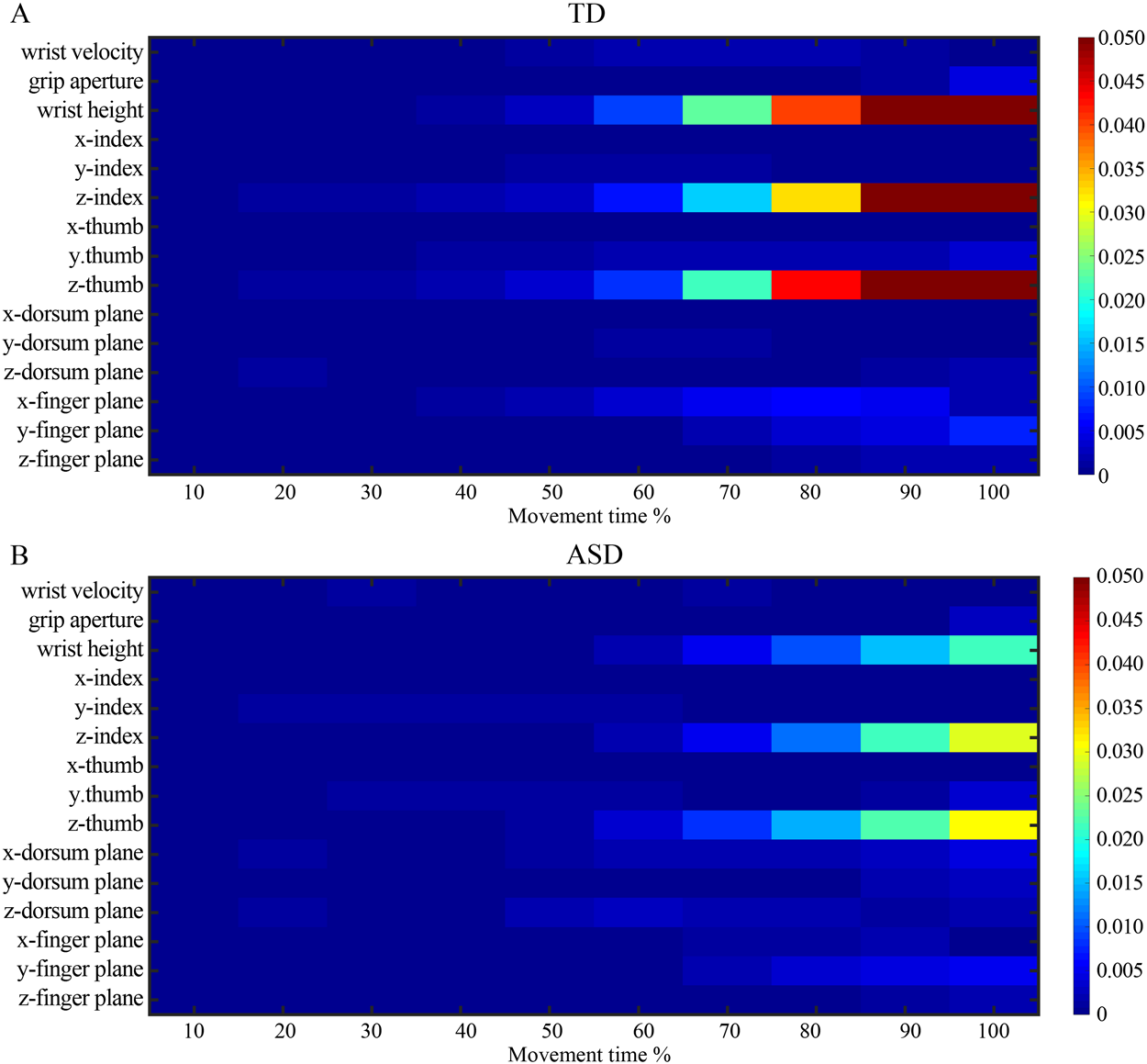
Discriminative power of kinematic features in TD (**A**) and ASD (**B**) group in prospective control of self-actions. The heatmaps show a graphical representation of Fisher scores of kinematic features over time. The higher the Fisher score, the greater the ability of a kinematic feature to discriminate between self-action plans. To allow comparison between groups, Fisher scores were normalized by dividing each score by the sum of all scores obtained in both groups.

To identify which kinematic features drove the classifier and evaluate the discriminative power of each kinematic feature over time, we next computed Fisher scores (see Data Analysis section). Fisher scores provide a measure of distance between data points in different classes of action. The higher the Fisher score, the greater the ability of a kinematic feature to discriminate between forthcoming actions. Figs 2A,B provide an overall view of the discriminative power of kinematic features in TD and ASD groups respectively. Visual inspection of the matrix revealed similar patterns of modulation in ASD and TD children. Specifically, in both groups, the specification of wrist height, index, and thumb height evolved gradually as the movement unfolded.

**Figure 3:**
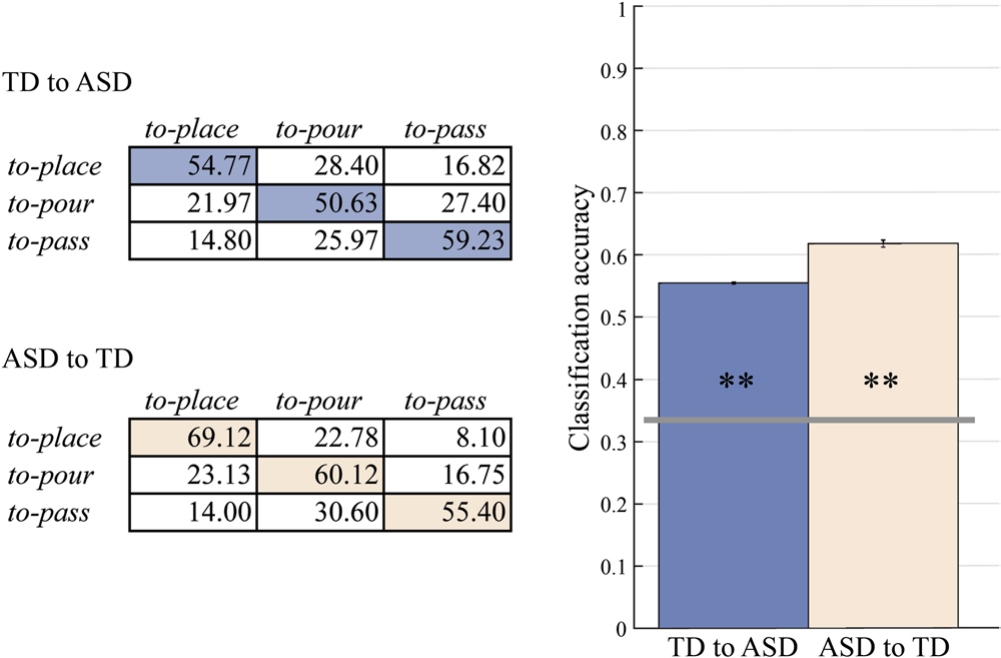
Cross-classification results for prospective control of self-actions. Confusion matrices and classification accuracies for a SVM classifier trained on TD and tested on ASD group (TD to ASD) and for a SVM classifier trained on ASD and tested on TD group (ASD to TD). The classification accuracy exceeded chance level (0.33; grey horizontal line) in both TD to ASD and ASD to TD cross-classifications (empirical p_s_ after 500 permutations = 0.002). Asterisks inside bars indicate significant differences from chance level classification (**p < 0.01).

To evaluate this impression and obtain quantitative evidence for similarity, in a subsequent analysis, we exploited an extension of the classification approach known as multivariate cross-classification^14,15^. Cross-classification requires that a classifier is trained on data from one group (or condition) and tested on data of another. The cross-classification approach provides a direct measure of the similarity between the patterns underlying the two groups (or conditions). Following this logic, we trained the SVM classifier on one group (e.g., TD) and then tested it on its ability to classify the actions of the other group (e.g., ASD). Classifier performance was well above chance level (TD to ASD: mean *accuracy* = 0.549, 95% CI = [0.539, 0.558], empirical *p* = 0.002; ASD to TD: mean *accuracy* = 0.616, 95% CI = [0.605, 0.629], empirical *p* = 0.002) (Fig. 3). This result corroborates the idea that ASD and TD children used similar prospective control strategies to accommodate subsequent self-actions.

### Prospective control of other-actions

To verify whether ASD and TD children altered their initial grasp in anticipation of the co-actor’s forthcoming action, we trained a SVM classifier to distinguish between *pass-to-place* and *pass-to-pour* actions. As for self-actions, for each group, classification performance was computed as the resulting average of a leave-one-subject-out cross-validation procedure. To test whether classification accuracy significantly exceeded chance level, we randomly permuted the class labels ‘*pass-to-place*’ and ‘*pass-to-place*’ (500 permutations) and recomputed the SVMs *classification accuracy* after each permutation.

**Figure 4:**
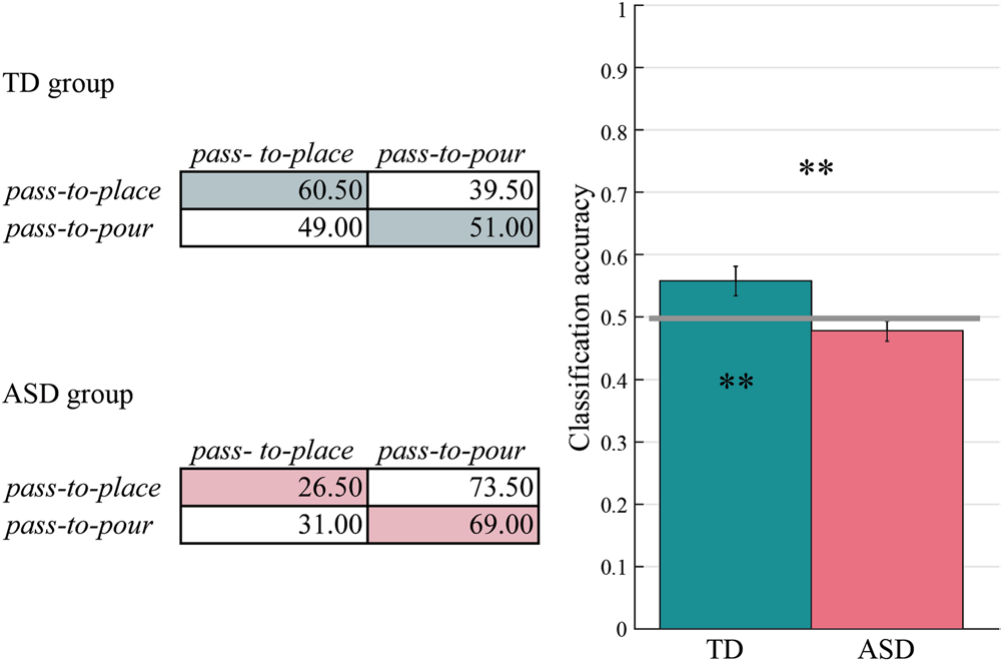
Prospective control of other-actions. Confusion matrices and classification accuracies for TD and ASD group. The classification accuracy exceeded chance level (0.50; grey horizontal line) only in TD group (empirical *p* after 500 permutations = 0.01) and was significantly lower in ASD compared to TD group (p < 0.01). Asterisks inside bars indicate significant differences from chance level classification. Asterisks outside bars indicate significant differences between groups (**p < 0.01).

The classifier performed above chance (0.50) in the TD group (mean = 0.558, 95% CI = [0.508, 0.607], empirical p = 0.01), but not in the ASD group (mean = 0.478, 95% CI = [0.444, 0.510], t_19_ = 3.439, p = 0.510). The classification accuracy for the ASD group was also significantly lower compared to the TD group (t_38_ = −2.807, p < 0.01). Figure 4 shows confusion matrices and *classification accuracies* for the two groups.

**Figure 5:**
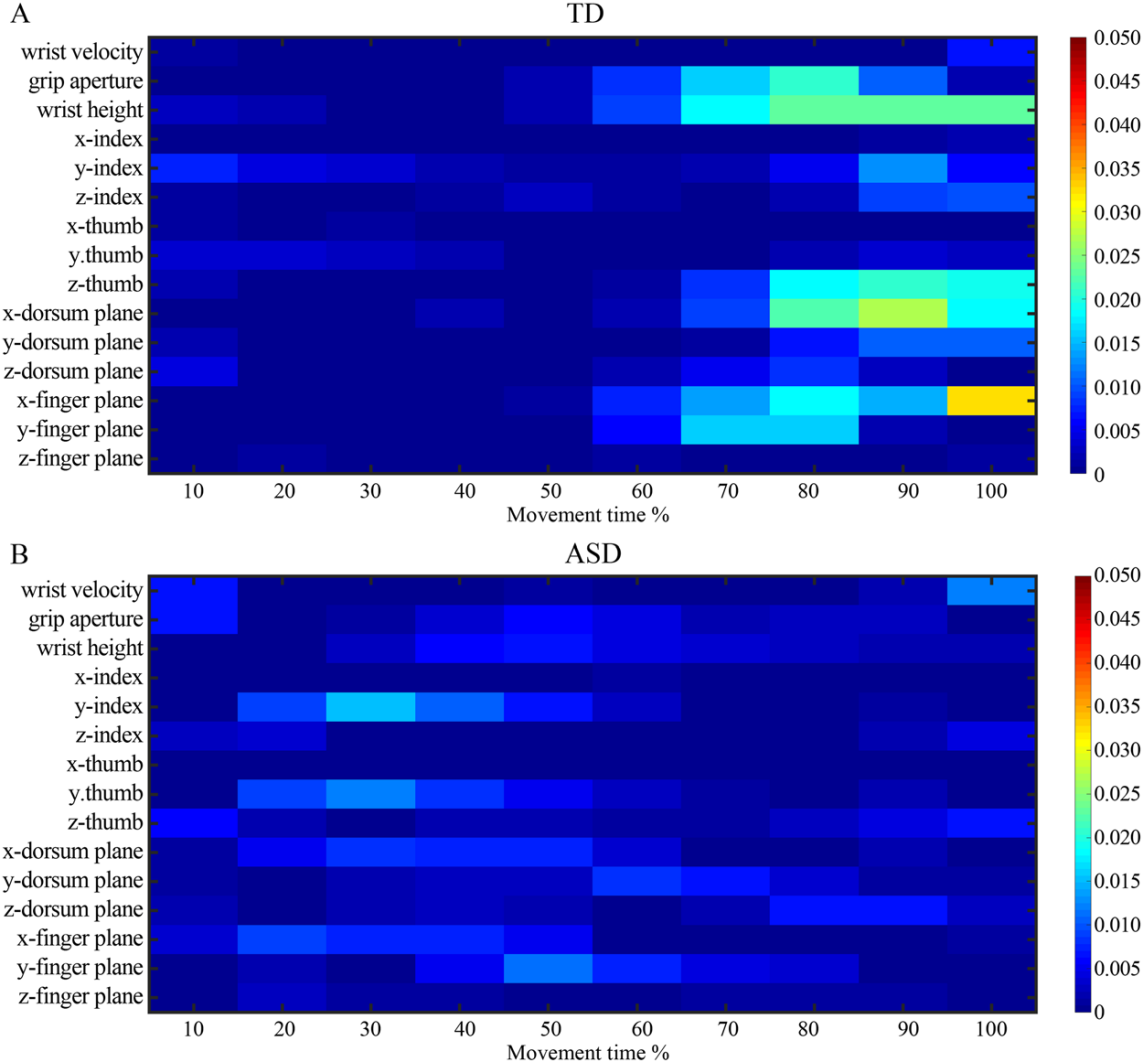
Discriminative power of kinematic features in TD (**A**) and ASD (**B**) group in prospective control of other-actions. The heatmap shows a graphical representation of Fisher scores of kinematic features over time. The higher the Fisher score, the greater the ability of a kinematic feature to discriminate between other-action plans. To allow comparison between groups, Fisher scores were normalized by dividing each score by the sum of all scores obtained in both groups.

We next computed Fisher scores to evaluate the discriminative power of each kinematic feature over time. In the TD group, as one would expect, the specification of different parameters of movement increased as the hand approached the object. In the ASD group, the specification of diverse aspects of movements appeared markedly attenuated, with only a few movement features showing an early, but not late, specification (Fig. 5).

**Figure 6:**
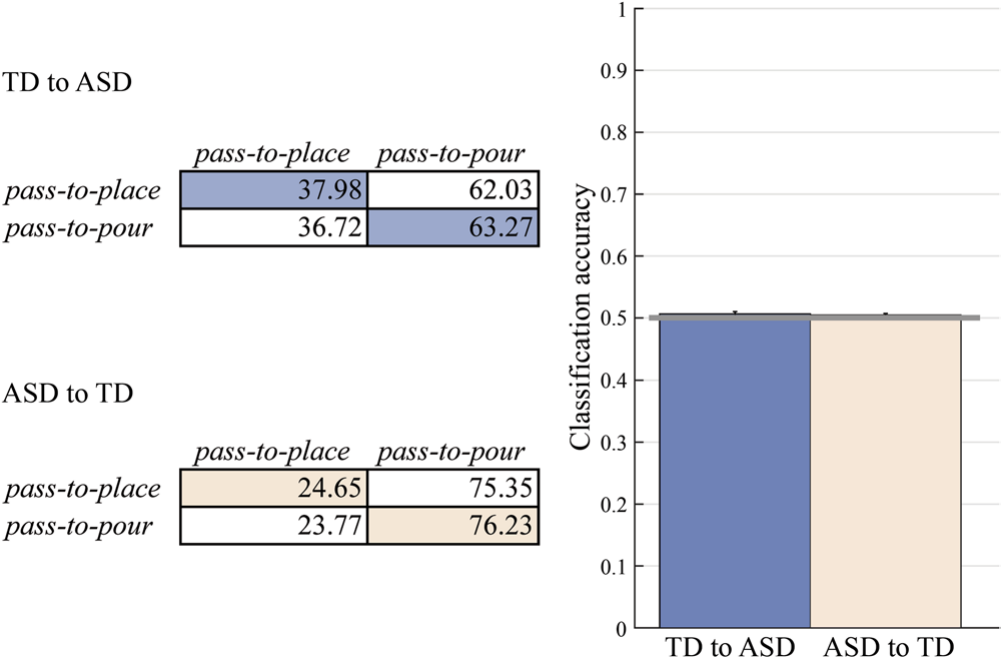
Cross-classification results for prospective control of other-actions. Confusion matrices and classification accuracies for a SVM classifier trained on TD and tested on ASD group (TD to ASD) and for a SVM classifier trained on ASD and tested on TD group (ASD to TD). The classification accuracy did not exceed chance level (0.50; grey horizontal line) in neither of the two cross-classifications.

Confirming this impression, cross-classification analysis was unsuccessful both when the classifier was trained on TD data and tested on ASD data (TD to ASD, mean *accuracy* = 0.506, 95% CI = [0.498, 0.515], empirical *p* = 0.305) and when it was trained on ASD data and tested on TD data (ASD to TD, mean *accuracy* = 0.504, 95% CI = [0.499, 0.510], empirical *p* = 0.621) (Fig. 6).

### Idiosyncrasy of movement patterns

The reduced to absent kinematic modulation observed in the ASD group may stem from different phenomena at the single-subject level. First, the observed group effect may reflect increased noise in the motor system^19^. Second, the effect may be due to a reduced degree of prospective control operating within each individual in the ASD group. Alternatively, it may reflect idiosyncrasies in the individual patterns of modulation, i.e., patterns that differ from one individual to another. On this account, the reduced pattern of modulation at the group level would result from misalignment of control strategies at the individual level causing a ‘regression to the mean effect’.

**Figure 7:**
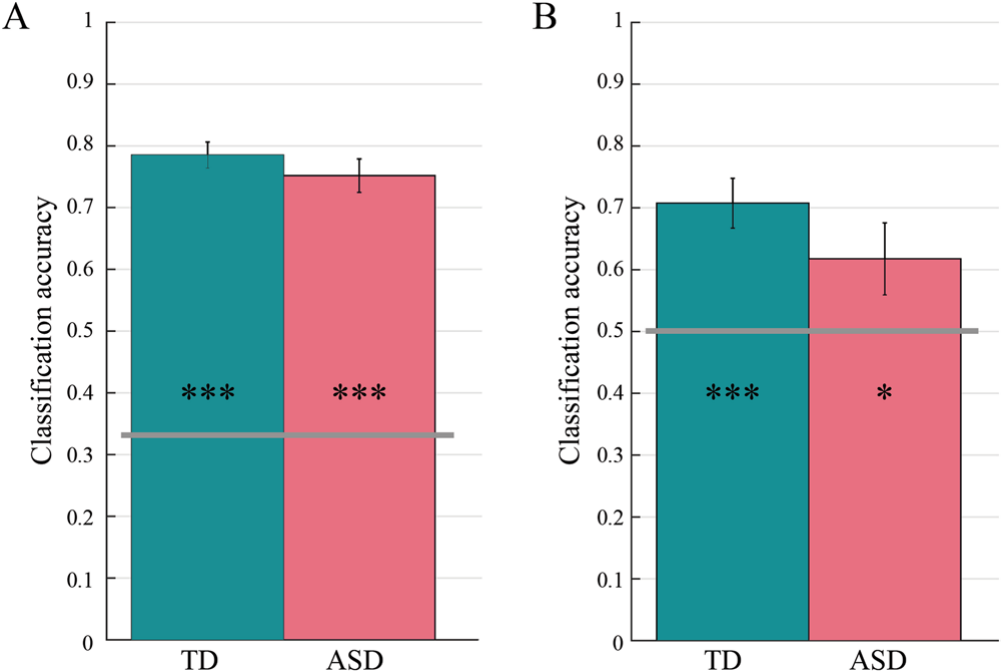
Individual classification performance for prospective control of self-actions (panel A) and other-actions (panel B). Classification accuracies obtained by averaging, separately for each group, results of 20 SVM classifiers (1 for each child). For prospective control of both self- (**A**) and other-actions (**B**), the classification accuracy exceeded chance level (grey horizontal lines) in both groups, with no significant differences between TD and ASD children. Asterisks inside bars indicate significant differences from chance level classification (***p < 0.001; *p < 0.05).

In order to test this possibility, we trained SVM classifiers to distinguish *place*, *pour* and *pass* actions separately for each child in the TD and ASD group. Individual classification performance was computed using a leave-one-trial out cross-validation procedure and quantified as the resulting *classification accuracy*. Individual classification performance exceeded chance level (0.33) in both the TD group (mean = 0.785, 95% CI = [0.741, 0.829], t_19_ = 21.515, p < 0.001) and the ASD group (mean = 0.755, 95% CI = [0.700, 0.810], t_19_ = 16.229, p < 0.001). No difference was observed between ASD and TD children (t_38_ = −0.891, p = 0.378), attesting comparable levels of modulation at the individual level (Fig. 7).

Notably, training SVM classifiers to distinguish *pass-to-place* and *pass-to-pour* actions separately for each child led to a similar pattern of results. Individual classification performance exceeded chance level (0.50) in both the TD group (mean = 0.708, 95% CI = [0.623, 0.792], t_19_ = 5.152, p < 0.001) and the ASD group (mean = 0.618, 95% CI = [0.517, 0.740], t_19_ = 2.016, p < 0.05), with no difference in classification accuracy between ASD and TD children; t_38_ = −1.270, p = 0.218). This indicates that the apparent absence of prospective motor control in anticipation of other-actions at the group level resulted entirely from misalignment of individual strategies.

### Relationship to symptoms and cognitive functions

To explore the possibility that prospective motor control in ASD is related to symptoms severity, we correlated self- and other-actions individual SVM classification accuracies with ADOS-2 and ADI-R total scores. Classification accuracies were also correlated with executive functions as measured by the Tower of London (ToL) test and with intelligent quotient as measured by the Full-Scale IQ. For each pair of variables, statistical significance was assessed with a non-parametric permutation test (1000 permutations). None of the correlations approached significance (*p*_*s*_ ranging from 0.178 to 0.834).

## Discussion

We developed a multi-level classification strategy to comprehensively test the hypothesis of disturbances of *prospective motor control* in children with autism spectrum disorder.

We found both similarities and dissimilarities in the prospective control strategies of ASD and TD children. For self-actions, although tailoring to the onward action was overall less pronounced in the ASD group than in the TD group, children in the two groups exhibited similar patterns of modulation. No such similarity was apparent for other-actions. Observing the heatmap in Fig. 5, one might be inclined to conclude that the kinematics of the movements conducted by children with ASD did no show changes in anticipation of the actions of the partner. However, this conclusion overlooks the heterogeneity of autistic movement patterns. When analyzed at the individual-level, the kinematics of ASD and control children showed comparable levels of modulation for both self- and other-actions. This suggests that the reduced to absent modulation at the group-level resulted from misalignment of individual control strategies rather than from a lack of control strategies in individuals with ASD.

Previous approaches investigating motor control in ASD only extrapolated group-level patterns, with limited success in capturing individual motor variability^18,20,21^. A critical advance of our study is to show that group-level patterns can obscure the heterogeneity of individual strategies in motor control. The implication of this finding is that statistics that average ASD individual movement profiles within a group may fail to capture a distinctive trait of ASD motor performance. That possibly accounts for discrepant findings in previous studies.

Future studies are necessary to understand the mechanisms that give rise to the idiosyncrasy of motor patterns. While there is a general consensus that individuality exists in motor patterns in both typical and atypical populations, how it arises and whether it reflects neural structure are still sources of debate^22^. In the present study, we observed no correlation with diagnostic tests of ASD. This suggests distinct movement profiles within otherwise similar diagnostic profiles. It will be important for future studies to determine whether individual movement profiles correlate with individual differences in the function and organization of the cortical grasping network. Cattaneo *et al*.^21^ report failure of predictive muscle activation during execution of a sequential grasping task (although Pascolo and Cattarinussi^23^ have recently failed to replicate this finding). Under the assumption that anticipatory muscle activation is an index of prospective motor control, we would expect the preparatory muscle activity to correlate with the degree of tailoring kinematics to the onward action at the individual level.

Disturbances of development in systems that program timing, serial coordination and prospective control of movements have been proposed to be at the origin of social isolation, socio-emotional and cognitive delay in ASD^7^. Whilst the current results provide no direct evidence to support this idea, they indicate that children with ASD demonstrate divergent, idiosyncratic patterns in anticipation of others’ actions. The consequences of this can be far-reaching. Kinematic similarity is thought to be important for the perception, prediction, and interpretation of others’ movements^24^. Moving with different kinematics, typical and autistic individuals may experience reciprocal difficulties in social interaction. Moreover, because each atypical movement pattern is atypical in its own way, individuals with autism may also experience difficulties interacting with autistic partners whose movement patterns are dissimilar from their own. These predictions can be tested in future studies by investigating social interaction in TD-ASD and ASD-ASD dyads.

## Methods

### Participants

Twenty children with autism spectrum disorder (ASD group: 18 males) without accompanying intellectual impairment and 20 typically developing children (TD group: 16 males) were recruited from the Child Neuropsychiatry Unit of the IRCCS ‘Giannina Gaslini’ Hospital and primary schools in Genova. Groups were matched for age (TD mean ± SD = 9.5 ± 1.5 years.months; ASD = 9.8 ± 1.5 years.months; t_38_ = −0.665, p = 0.510), gender, Full Scale IQ as measured by the Wechsler Scale of Intelligence (WISC IV)^25^ (TD mean ± SD = 102.8 ± 9.4; ASD = 98.5 ± 11.1; t_38_ = 1.325, p = 0.193) and WISC IV subscales (verbal comprehension, perceptual reasoning, working memory and processing speed; Holm-Bonferroni corrected *p*_*s*_ ranging from 0.104 to 0.771; see Supplementary Table S1). The research protocol was approved by the local ethics committee (ASL3 Genovese) and was in accordance with the principles of the revised Helsinki Declaration^26^. Parents provided written informed consent after receiving a detailed description of the study.

Children with ASD were diagnosed according to DSM-5^27^ criteria. The Autism Diagnostic Observation Scale (ADOS-2)^28^ and Autism Diagnostic Interview-Revised (ADI-R)^29^ were administered by two experienced professionals. ASD children met the cut-off on total ADOS score (>=7) and on at least three out of four subscales of the ADI-R (see Supplementary Table S2). All ASD and TD children had normal or corrected-to-normal vision and were screened for exclusion criteria (pharmacological treatment, dyslexia, epilepsy, and any other neurological and psychiatric conditions). Both ASD and TD group were assessed for executive functions abilities by means of the Tower of London (TOL) test^30^. This test revealed no significant differences between TD and ASD children (TD mean ± SD = 29.35 ± 3.54; ASD = 29.35 ± 2.80; t_38_ = 0, p = 0.999). All but two of the children (one in the ASD group and one in the TD group) were right-handed according to the Edinburgh Handedness Inventory^31^.

### Stimuli and procedure

Children were tested individually in a quiet room. They were seated on a height-adjustable chair with their right elbow and wrist resting on a table (height = 64 cm; length = 100 cm; width = 60 cm). In order to guarantee a repeatable start position, they were asked to maintain their forearm in a pronated position with their right arm oriented in the parasagittal plane passing through the shoulder and their right hand in a semi-pronated position. They were asked to keep their thumb and index fingers closed in a pincer grip on a tape-marked point (at about 7 cm from table edge) on the working table. An open plastic bottle filled with water (base diameter = 5 cm; height = 18 cm; weight = 225 g) was positioned on the table at a distance of 44 cm from children’s midline. Throughout the entire experimental session, the same female experimenter (co-actor), sitting at the opposite side of the table, interacted with the children. Depending on conditions, one of two target objects was placed on the table: a box (height = 6 cm; diameter = 10 cm) or a glass (height = 10 cm; diameter = 6.5 cm). For *grasp-to-place* and *grasp-to-pour* trials, the target object was located 19 cm away from the bottle. For *grasp-to-pass* trials, the target object was located closer to the co-actor’s right hand, 43.5 cm away from the bottle. Children performed a series of 12 consecutive grasps for each condition; they made a total of 48 movements. In each trial, children were asked to perform at a natural speed after an auditory tone. During *grasp-to-place* and *grasp-to-pour* trials, the experimenter was asked to look down at the table, with her arms along the body. During *grasp-to-pass* trials, she was asked to look at the object, resting her right wrist on the table, with her thumb and index fingers closed in a pincer grip on a tape-marked point on the working table. To avoid online influences of action perception on action production, the experimenter was instructed to start the movement only after the child had grasped the object.

The order of block presentation was pseudo-randomized across participants. Before each block, there were two practice trials to familiarize children with tasks. To avoid fatigue and lack of attention, children were given a two-minute pause at the end of each block. Testing required a single session of approximately 30 min per participant.

### Kinematics recording and data processing

A near-infrared camera motion capture system (frame rate = 100 Hz; Bonita Vicon Motion Systems Ltd, Oxford, UK) was used to track and record the reach-to-grasp kinematics. Six cameras were placed at a distance of 1.5–2 m from the working table. The child’s right hand was outfitted with 8 retro-reflective hemispheric markers (6.5 mm in diameter) placed on the metacarpal joint and the tip of the index and the little finger, the trapezium bone of the thumb, the radial aspect of the wrist and the center of the hand dorsum. After the data collection, each trial was individually inspected for correct marker identification and then run through a 6 Hz low-pass Butterworth filter. For data processing, a custom software (Matlab; MathWorks, Natick, MA) was used to compute two sets of kinematic variables.

The first set of variables, expressed with respect to the original frame of reference (i.e., the frame of reference of the motion capture system, termed as the global frame of reference), included:

- *wrist velocity*, defined as the module of the velocity of the wrist marker (mm/sec);
- *wrist height*, defined as the z-component of the wrist marker (mm);
- *grip aperture*, defined as the distance between the marker that was placed on the tip of the thumb and the marker placed on the tip of the index finger (mm).

To better characterize hand movements at the joint level, the second set of features was expressed with respect to a local frame of reference centered on the hand (i.e., F_local_; see^3^ for a detailed description of this frame of reference). This set of features included:

- *x-, y-, and z-thumb*, defined as x-, y- and z-coordinates for the thumb (mm);
- *x-, y-, and z-index*, defined as x-, y- and z-coordinates for the index finger (mm);
- *x-, y-, and z-finger plane*, defined as x-, y- and z-components of the thumb-index plane, i.e., the three-dimensional components of the vector that is orthogonal to the plane. This feature provides information about the abduction/adduction movement of the thumb and index finger, irrespective of the effects of wrist rotation and of finger flexion/extension;
- *x-, y-, and z-dorsum plane*, defined as x-, y- and z-components of the radius-phalanx plane. This feature provides information about the abduction, adduction, and rotation of the hand dorsum, irrespective of the rotation of the wrist.

All features were computed only considering the reach-to-grasp phase of the movement, i.e., from ‘reach onset’ (i.e., the first time point at which the wrist velocity crossed a 20 mm/s threshold and remained above it for longer than 100 ms) to ‘grasp offset’ (i.e., the time at which the wrist velocity dropped below a 20 mm/s threshold), at intervals of 1% of the normalized movement time.

### Data Analysis

For each group of participants, SVMs with Gaussian Kernel were used to solve two machine learning tasks: (i) classification of grasping movements followed by one of three onward self-actions (i.e. *place*, *pour*, and *pass*); (ii) classification of grasping movements followed by one of two onward other-actions (i.e. *pass-to-place* and *pass-to-pour*) (See Supplementary Material for more details about SVM models). For both tasks, the *classification accuracy* was used as a measure of SVM classification performance. For group-level analyses, classification accuracies were computed using a leave-one-subject-out cross-validation scheme and submitted to independent-samples t-tests for comparison between TD and ASD. Shapiro-Wilk tests and Levene’s test were performed to verify normality of the distributions and homogeneity of variance (all *p*_s_ above 0.200).

For individual-level analyses, classification accuracies were computed from a leave-one-trial-out cross-validation scheme. One sample t-tests were used to test classification accuracies against chance level. Normality of distribution was verified with Shapiro-Wilk test of normality (all p_s_ above 0.200).

To evaluate the discriminative power of each kinematic feature in the planning of both self- and other-actions, we calculated for each participant and each kinematic feature the Fisher score. Given a dataset $\{({x}_{i},{y}_{i})\}{\,}_{i=1}^{n}$ where ${x}_{i}\in {{\mathbb{R}}}^{d}\,{\rm{and}}\,{y}_{i}\in \{1,2,\,\ldots ,\,{\rm{c}}\}$, the Fisher score for the k-th feature is defined as:

$$Fisher\,score\,({x}^{k})=\,\frac{{{\sum }^{}}_{j=1}^{c}\,{p}_{j}({\mu }_{j}^{k}-{\mu }^{k})}{{{\sum }^{}}_{j=1}^{c}\,{p}_{j}{({{\rm{\sigma }}}_{j}^{k})}^{2}}$$

Where ${\mu }_{j}^{k}$ and ${{\rm{\sigma }}}_{j}^{k}$ are the mean and the standard deviation of the *j*-th feature, while μ^*k*^ and *p*_*j*_ denote the mean of the whole dataset corresponding to the *k*-th feature and the class prior probability, respectively.

In order to allow comparisons between TD and ASD children, Fisher scores of each feature were then normalized by dividing each score by the sum of all scores obtained in both groups.

## Electronic supplementary material

Supplementary methods

Video S1 - GraspToPour - TD

Video S2 - GraspToPour - ASD

Video S3 - GraspToPlace - TD

Video S4 - GraspToPlace - ASD

Video S5 - PassToPour - TD

Video S6 - PassToPour - ASD

Video S7 - PassToPlace - TD

Video S8 - PassToPlace - ASD
