# Supplementary material for: Prospective motor control obeys to idiosyncratic strategies in autism
Source: Sci Rep. 2018 Sep 12;8:13717. doi: 10.1038/s41598-018-31479-2 (PMC6135837; doi:10.1038/s41598-018-31479-2)
Supplement: Supplementary file 1 — Supplementary methods [file 41598_2018_31479_MOESM1_ESM.pdf]

Prospective motor control obeys to idiosyncratic strategies in autism

Andrea Cavallo[1,2], Luca Romeo[3,4], Caterina Ansuini[2], Jessica Podda[2], Francesca Battaglia[5], Edvige Veneselli[5], Massimiliano Pontil[3,6] & Cristina Becchio*[2,1]

[1] Department of Psychology, University of Torino, Italy

[2] C'MoN Unit, Fondazione Istituto Italiano di Tecnologia, Genova, Italy

[3] Computational Statistics and Machine Learning, Fondazione Istituto Italiano di Tecnologia, Genova, Italy

[4] Dipartimento di Ingegneria dell'Informazione, Università Politecnica delle Marche, Italy

[5] Child Neuropsychiatric Unit, G. Gaslini Institute, Genoa, Italy

[6] Department of Computer Science, University College London, U.K.

Supplementary Material

SVM Model

SVM is a non-probabilistic kernel-based decision machine that leads to a sparse solution. This implies that predictions for new inputs depend only on the kernel function evaluated at a subset of the training data points, called *support vectors*. The determination of the model parameters corresponds to a convex optimization problem, and so any local solution coincides with a global optimum. These properties allow to reduce the computational time while increasing the algorithm performance. SVMs have been used for solving object recognition tasks[1–3], regression and time series prediction applications[4–6], and novelty detection problems[7–9].

Given the training data set composed by M input vectors $x_{1}, \ldots, x_{m}$, with corresponding target values $y_{1}, \ldots, y_{m}$ where $y_i \in \{-1, 1\}$, new datapoints x can be classified according to the sign of $f(x)$. In the case of linear function f, the model takes the form:

$$f(x) = \omega^{t} \varphi(x) + b \qquad \text{(Eq. 1)}$$

where $\varphi(x)$ denotes a fixed feature mapping, ω and b are the model parameters. The SVM aims to choose the decision boundary in order to maximize the margin, which is defined to be the smallest distance between the decision boundary and any of the samples. Since the class-conditional distributions may overlap, the exact separation of the data can lead to poor generalization. Thus, the introduction of the slack variables $\varepsilon_i \geq 0$ where $i = 1, \ldots, M$ allows some of the training set data points to be misclassified, and then to overlap class distribution. Then, a SVM problem formulation can be seen as the optimization of the "soft margin" loss function[10].

$$\text{minimize } \frac{1}{2} |\omega|^2 + C \sum_{i=1}^{M} \varepsilon_i \qquad \text{(Eq. 2)}$$

subject to $y_i(\omega^T \varphi(x) + b) \geq 1 - \varepsilon_i$

$\varepsilon_i \geq 0$

The parameter $C > 0$ is known as box constraint and controls the trade-off between the slack variable penalty and the margin. The procedure for solving Eq. 2 is to construct a Lagrange function from the objective function and the corresponding constraints, by introducing a dual set of variables. The key observation is that the Lagrangian solution leads to the dual representation of the maximum margin problem in which we maximize:

$$\tilde{L}(a) = \sum_{i=1}^{M} a_i - \frac{1}{2}\sum_{i,j=1}^{M} a_i a_j y_i y_j K(x_i, x_j) \qquad \text{(Eq. 3)}$$

under constraints $\sum_{i=1}^{M} a_i\, y_i = 0$ and $0 \leq a_i \leq C$, where a_i are the Lagrange multipliers and $K(x_i, x_j)$ is the kernel function defined by the Gaussian kernel:

$$K(x_i, x_j) = exp\left(-\gamma \left|x_i - x_j\right|^2\right) \qquad \text{(Eq. 4)}$$

where γ is the kernel scale parameter. The optimization of Eq. 3 takes the form of a quadratic programming problem where the computational complexity in the dual problem (see Eq. 3) depends on the number of samples (i.e., *M*). If $(\hat{a}_i)$ is the solution of the dual problem in Eq. 3, the prediction of new data points can be expressed in terms of the parameters and the kernel function as follows:

$$f(x) = \sum_{i=1}^{M} \hat{a}_i y_i K(x, x_i) + b \qquad \text{(Eq. 5)}$$

Note that any data point for which $\hat{a}_i = 0$ does not contribute to the prediction (see Eq. 5), while the remaining data points constitute the support vectors. SVM hyperparameters (i.e, γ and C) used to solve the machine learning tasks (i.e. classification of grasping movements followed by i. self-actions and ii. other-actions) were chosen in order to minimize the *validation error* within the leave-one-subject-out cross-validation.

Supplementary Table S1. WISC-IV scores for participants of TD group and ASD group

WISC IV scores	TD group		ASD group	
	Mean	SD	Mean	SD
Full scale IQ	102.8	9.4	98.5	11.1
Verbal comprehension	105.3	9.6	98.3	14.6
Perceptual reasoning	107.6	11.4	108.6	11.2
Working memory	102.4	10.8	94.6	10.5
Processing speed	94.4	14.5	89.2	14.9

Note: TD = Typically Developing; ASD = Autism Spectrum Disorder;
SD = Standard Deviation

Supplementary Table S2. ADOS-2 and ADI-R scores for participants of the ASD group.

Participant	ADOS-2			ADI-R				
	Total score	SA	RRB	Total score	A)	B)	C)	D)
001	*8*	7	1	25	*12*	*8*	*3*	*2*
002	*8*	6	2	29	*10*	*10*	*8*	*1*
003	*8*	6	2	30	*12*	*9*	*7*	*2*
004	*8*	6	2	30	*12*	*9*	*7*	*2*
005	*8*	6	2	28	*10*	*11*	*5*	*2*
006	*9*	8	1	25	*10*	*8*	*5*	*2*
007	*8*	6	2	28	*11*	*9*	*4*	*4*
008	*8*	7	1	31	8	*17*	*5*	*1*
009	*8*	7	1	49	*20*	*15*	*10*	*4*
010	*13*	11	2	21	9	*8*	*3*	*1*
011	*9*	8	1	41	*18*	*19*	*3*	*1*
012	*8*	7	1	30	*11*	*11*	*5*	*3*
013	*8*	7	1	24	*10*	*8*	*5*	*1*
014	*10*	8	2	29	*11*	*8*	*5*	*5*
015	*9*	8	1	32	*12*	*11*	*6*	*3*
016	*8*	7	1	24	*10*	*9*	*4*	*1*
017	*9*	8	1	24	*10*	7	*6*	*2*
018	*8*	7	1	25	*11*	*9*	*3*	*2*
019	*8*	6	2	24	*10*	5	*7*	*2*
020	*7*	6	1	27	*14*	3	*6*	*4*

Note: ADOS-2 (Autism Diagnostic Observation Scale 2) subtests: SA (Social Affect); RRB (Restricted and Repetitive Behaviors). Cut-off score for ADOS-2 Total Score (SA + RRB): (autism = 9; autism spectrum = 7). ADOS-2 Total score range (0–28). ADI-R (Autism Diagnostic Interview-Revised) subtests: A) Qualitative Abnormalities in Reciprocal Social Interaction (cut-off score = 10); B) Qualitative Abnormalities in Communication (cut-off score = 8); C) Restricted, Repetitive, and Stereotyped Patterns of Behavior (cut-off score = 3); D) Abnormality of Development Evident at or Before 36 Months (cut-off score = 1). ADI-R Total score range (0–78). The scores in Italics meet cut-off criteria.

Supplementary Figure S1. Frame of a grasp-to-pour movement performed by a child of the TD group (100% of movement time)

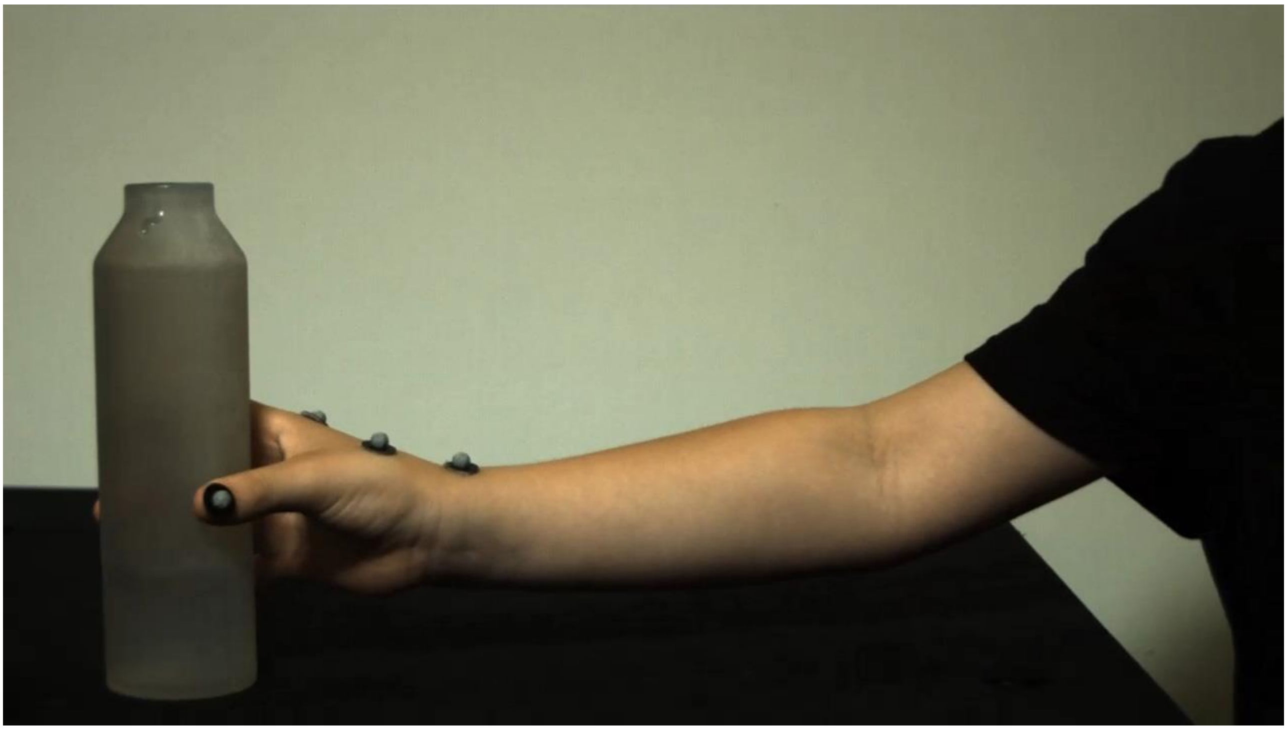

Supplementary Figure S2. Frame of a grasp-to-pour movement performed by a child of the ASD group (100% of movement time)

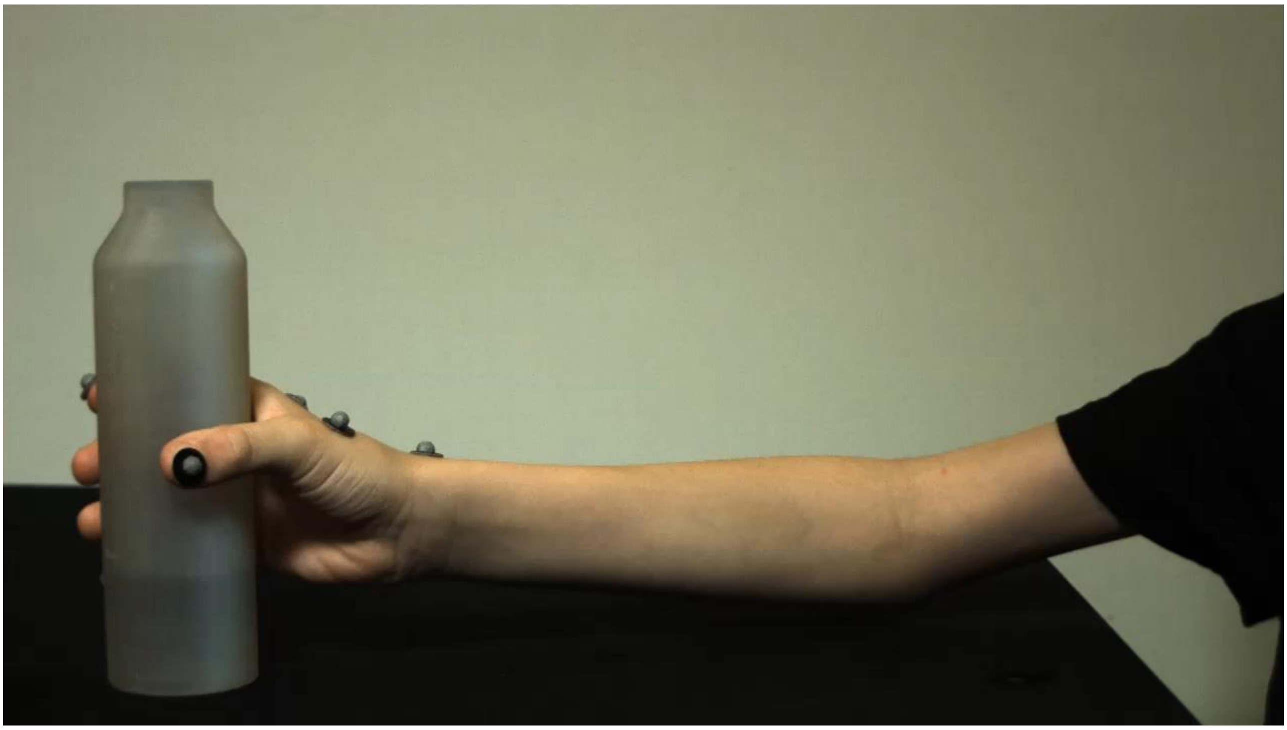

Supplementary Figure S3. Frame of a grasp-to-place movement performed by a child of the TD group (100% of movement time)

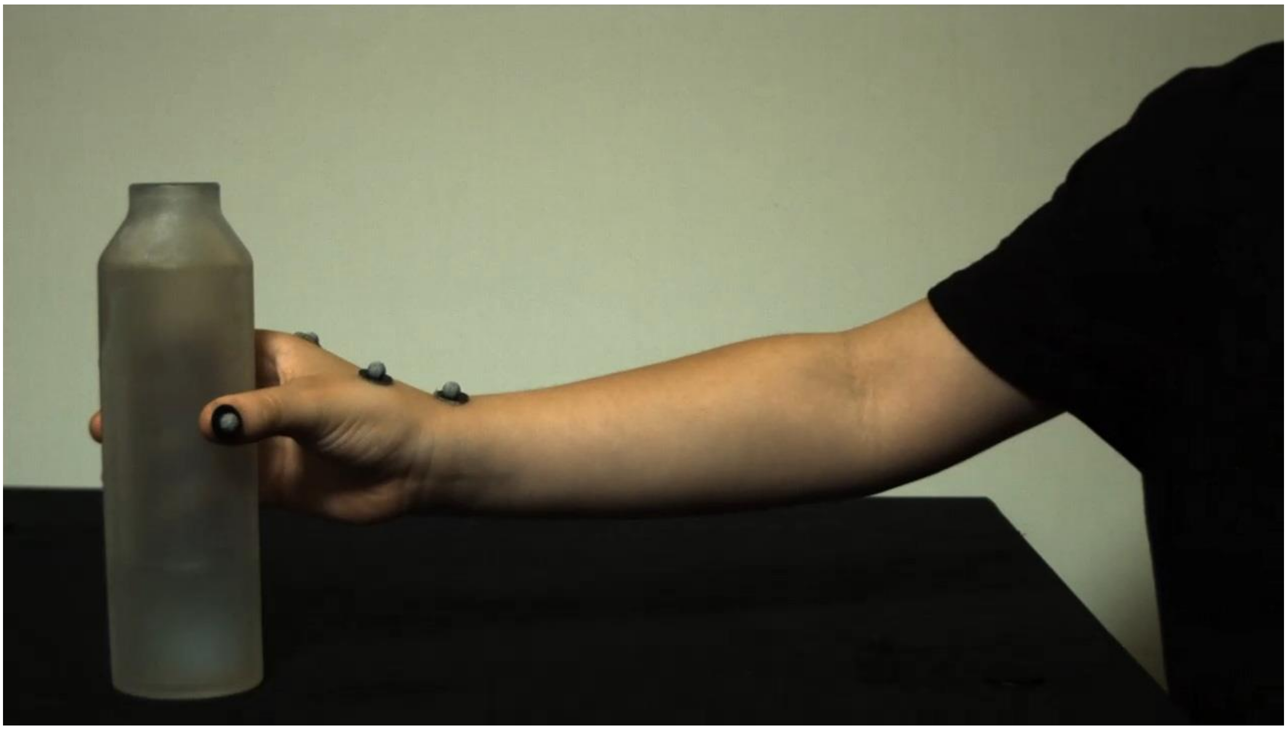

Supplementary Figure S4. Frame of a grasp-to-place movement performed by a child of the ASD group (100% of movement time)

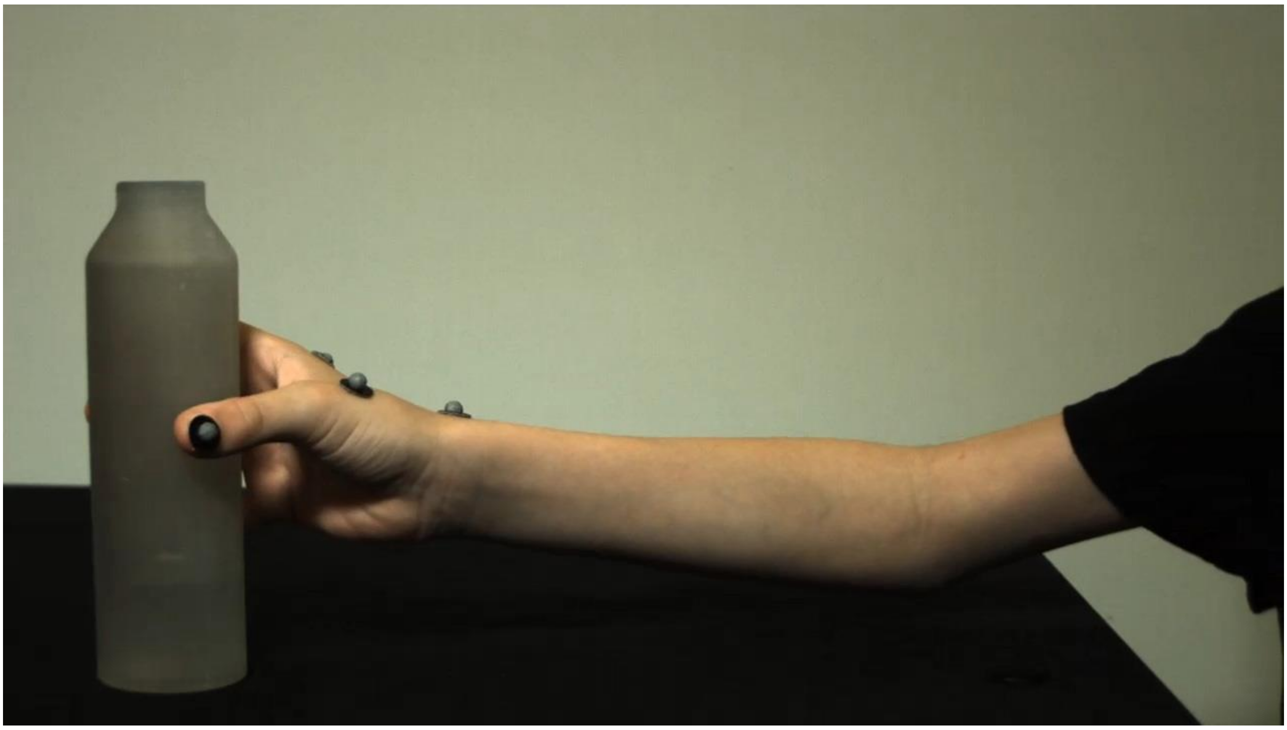

Supplementary Figure S5. Frame of a pass-to-pour movement performed by a child of the TD group (100% of movement time)

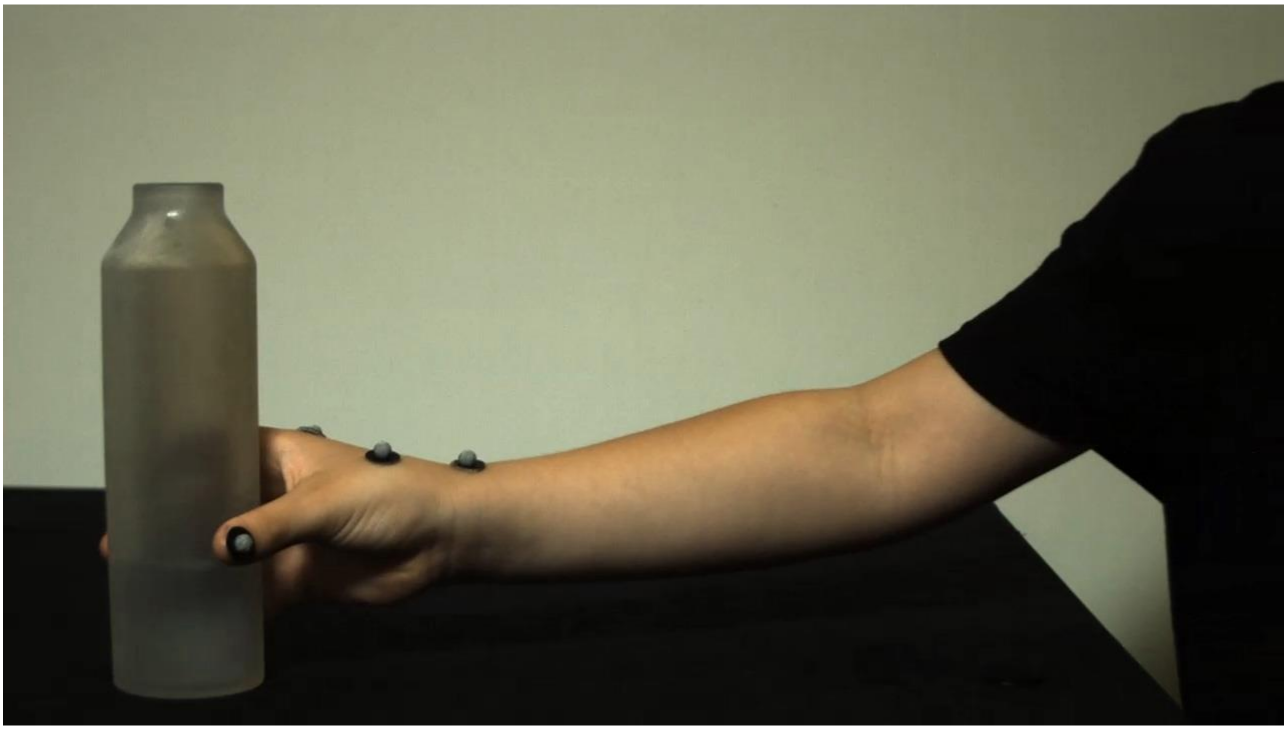

Supplementary Figure S6. Frame of a pass-to-pour movement performed by a child of the ASD group (100% of movement time)

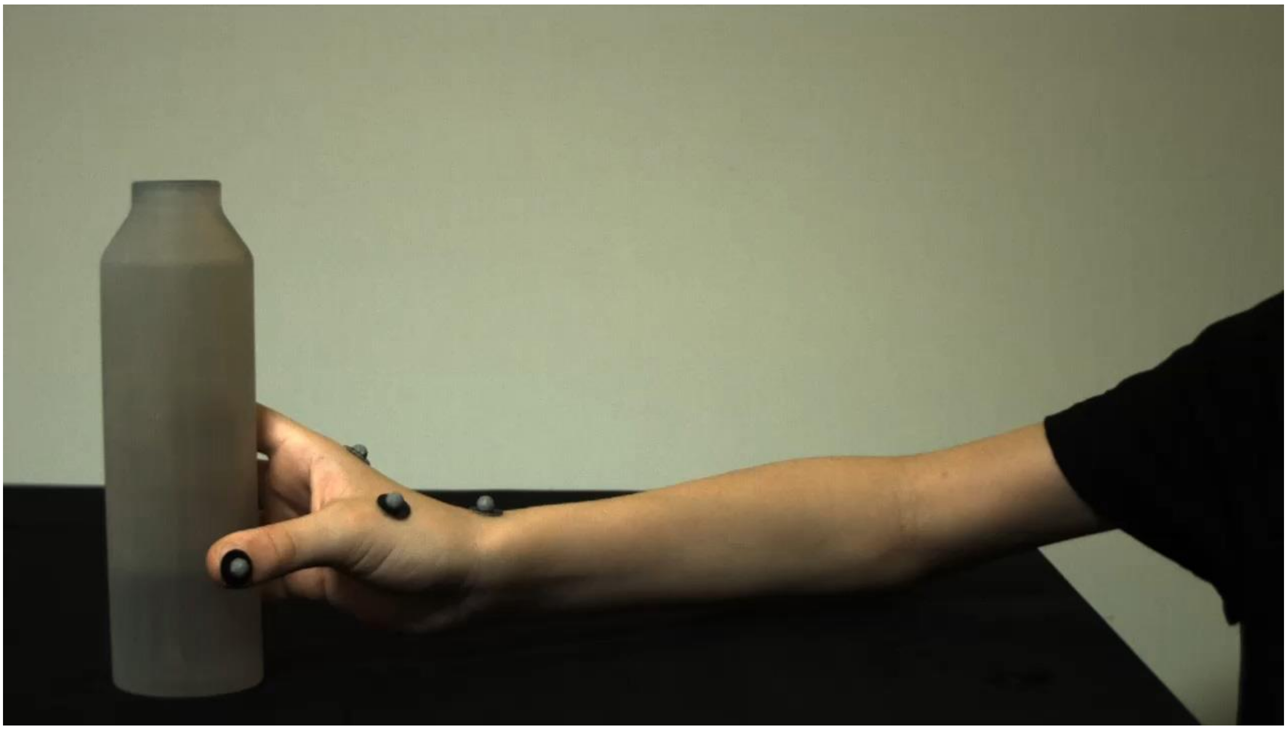

Supplementary Figure S7. Frame of a pass-to-place movement performed by a child of the TD group (100% of movement time)

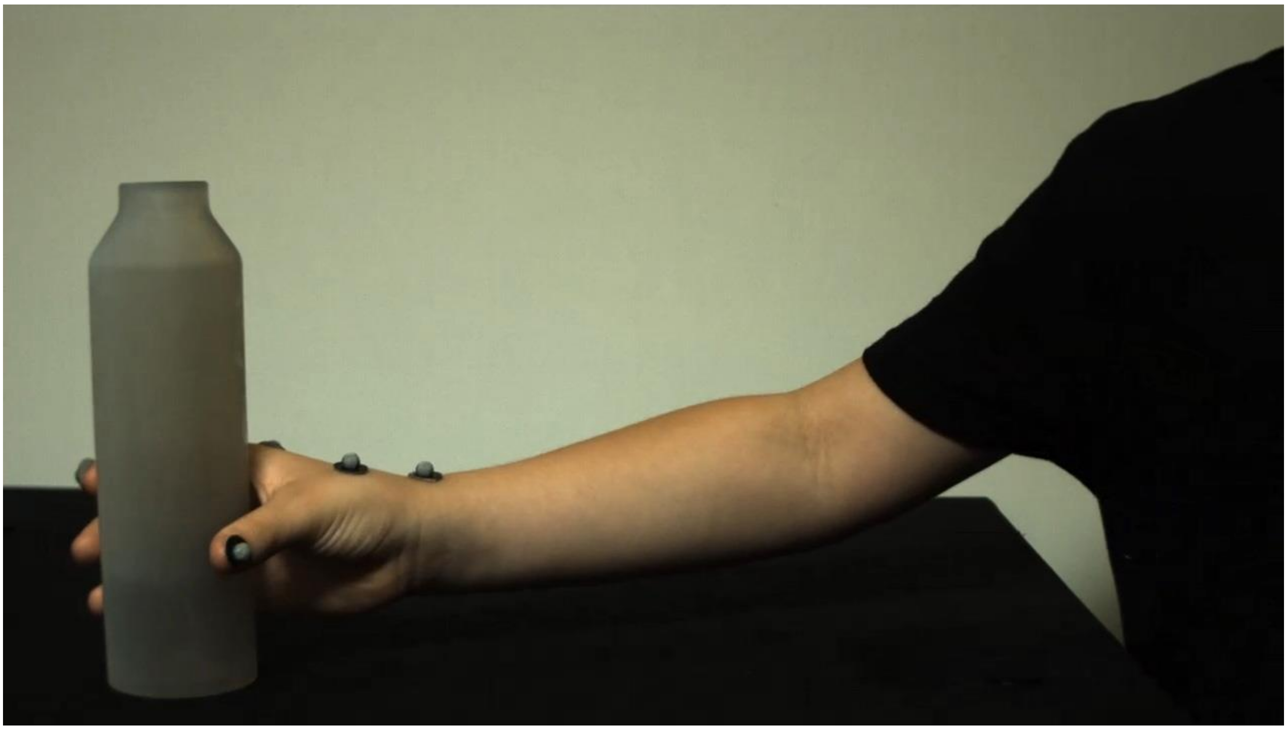

Supplementary Figure S8. Frame of a pass-to-place movement performed by a child of the ASD group (100% of movement time)

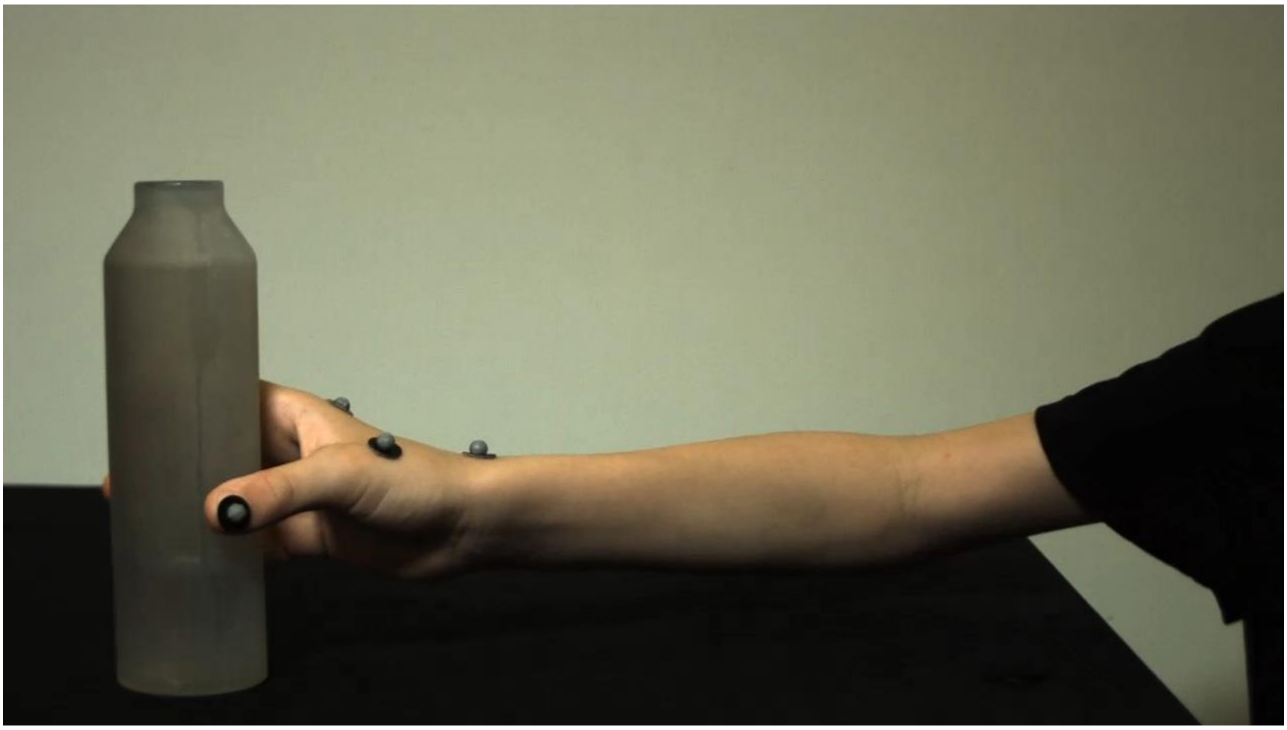

References

1. Chapelle, O., Haffner, P. & Vapnik, V. N. Support vector machines for histogram-based image classification. *Neural Networks, IEEE Trans.* **10,** 1055–1064 (1999).

2. Schölkopf, B., Burges, C. & Vapnik, V. Incorporating invariances in support vector learning machines. in *Lecture Notes in Computer Science (including subseries Lecture Notes in Artificial Intelligence and Lecture Notes in Bioinformatics)* **1112 LNCS,** 47–52 (1996).

3. Blanz, V. *et al.* Comparison of view-based object recognition algorithms using realistic 3D models. *Icann* **1112,** 251–256 (1996).

4. Muller, K. *et al.* Predicting time series with support vector machines. in *Artificial Neural Networks—ICANN'97* **1327,** 999–1004 (1997).

5. Stitson, M., Gammerman, A. & Vapnik, V. Support Vector regression with ANOVA Decomposition Kernels. *Support vector* 1–22 (1999).

6. Drucker, H., Burges, C. J. C., Kaufman, L., Smola, A. & Vapnik, V. Support vector regression machines. *Adv. Neural Inf. Process. Syst.* **9,** 155–161 (1997).

7. Schölkopf, B., Williamson, R., Smola, A., Shawe-Taylor, J. & Platt, J. Support Vector Method for Novelty Detection. *Nips* **12,** 582–588 (1999).

8. Hoffmann, H. Kernel PCA for novelty detection. *Pattern Recognit.* **40,** 863–874 (2007).

9. Gardner, A., Krieger, A., Vachtsevanos, G. & Litt, B. One-class novelty detection for seizure analysis from intracranial EEG. *J. Mach. Learn. Res.* **7,** 1025–1044 (2006).

10. Cortes, C. & Vapnik, V. Support-vector networks. *Mach. Learn.* **20,** 273–297 (1995).
